# The Gestalt of functioning in autism revisited: First revision of the International Classification of Functioning, Disability and Health Core Sets

**DOI:** 10.1177/13623613241228896

**Published:** 2024-02-13

**Authors:** Sven Bölte, Lovisa Alehagen, Melissa H Black, John Hasslinger, Elina Wessman, Karl Lundin Remnélius, Peter B Marschik, Emily D’Arcy, Susanna Crowson, Megan Freeth, Andreas Seidel, Sonya Girdler, Eric Zander

**Affiliations:** 1 Karolinska Institutet, Sweden; 2 Region Stockholm, Sweden; 3 Curtin University, Australia; 4 University Medical Center Göttingen and Leibniz Science Campus Primate Cognition, Germany; 5 Medical University of Graz, Austria; 6 University of Sheffield, UK; 7 University of Applied Sciences Nordhausen, Germany

**Keywords:** adolescents, adults, assessment, autism, children, Core Sets, functioning, International Classification of Functioning, Disability and Health, participation, revision, validation, World Health Organization

## Abstract

Functioning is a construct capturing how an individual’s engagement in everyday life emerges from the interaction between the individual and their environment. The World Health Organization’s International Classification of Functioning, Disability and Health (ICF) provides a biopsychosocial framework of functioning. Previously, the ICF was adapted for use in autism by developing Core Sets, a selection of ICF codes from the entire classification for specific conditions. Here, we present the first revision of the ICF of the Core sets for autism from a Delphi-like technique, based on evidence from Core Sets validation/linking studies, stakeholder feedback, and the development and piloting of the autism ICF Core Sets platform. Two ICF second-level codes were removed, and 12 were added to the comprehensive autism Core Set. The added codes reflect body functions in various sensory domains, fine hand use, and environmental factors. Changes were extensive for the age-appropriate brief Core Sets where ICF codes from the initial Core Sets were added or removed. The revisions conducted indicate a continued need for regularly updating Core Sets, based on empirical evidence and stakeholder involvement. We recommend the updated Core Sets for future use in autism research and practice in different age groups and contexts.

**Lay abstract:**

Autistic people experience individual strengths and challenges as well as barriers and facilitators in their environment. All of these factors contribute to how well autistic people can cope in everyday life, fulfill the roles they choose, and meet their needs. The World Health Organization has developed a system aiming to capture the many factors within people (like how someone thinks and feels) and outside of people (things around a person) that influence their daily living, called the International Classification of Functioning, Disability and Health. The International Classification of Functioning, Disability and Health can be used for different purposes in research and practice to assess people’s situations and plan support measures. Previously in 2019, the International Classification of Functioning, Disability and Health was adapted to autism by developing so-called Core Sets, which are shorter International Classification of Functioning, Disability and Health versions for use in specific conditions. Here, we present the first revisions of the International Classification of Functioning, Disability and Health Core Sets for autism, based on research, development results, and community feedback. Some factors influencing daily life for autistic people were added to the Core Sets, and other factors deemed less relevant were removed. Changes were also made in Core Sets designed for different age groups (0–5, 6–16, and ⩾17 years). Particularly, contents for sensory processing (like smell, touch, seeing, hearing) were added. We recommend these updated Core Sets for future use in autism research and practice. These changes to the Core Sets after 4 years indicate that there should be ongoing updates based on research and experience from practice and involvement of stakeholders.

## Introduction

Human science disciplines seek to describe and understand the essence and details of individuals’ physical characteristics, behaviors, beliefs, values, experiences, and living situations, as well as their inter-individual differences. Human science draws from biology, neuroscience, psychology, medicine, pedagogy, and sociology, their generic and specific models and explanations for human variability. These models drive how disciplines conceptualize issues, how professionals are socialized in their fields and engaged with their clients, and the paradigms underpinning research approaches in their fields. These disciplines are characterized by fundamental constructs, which guide their assessment practices, approaches, and interventions. Across disciplines, models and constructs are used as tools to understand human behaviors and outcomes.

Autism is a construct, and while many scientific disciplines are relevant to autism (Bölte & Richman, 2019), it has been traditionally defined by a medical model, where “deficient” behaviors (symptoms) within each individual cause low performance or productivity losses challenging social integration (impairment). The medical model of autism, enshrined in the leading diagnostic manuals *International Classification of Diseases-11* and *Diagnostic and Statistical Manual of Mental Disorders* (5th ed.; *DSM*-5) by the World Health Organization (WHO, 2019/2021) and American Psychiatric Association (APA, 2013), has dominated thinking in the field for decades, largely determining the focus of research, social policies, and support systems globally. However, recently this approach has been challenged, with calls from the neurodiversity movement, researchers, and professionals for a shift in thinking. While still recognizing the biological differences underpinning autism, the neurodiversity movement rejects the idea of disorder, calling for acknowledgment of the heterogeneity of autism and the negative impact of social barriers and stigma on functioning outcomes (Pellicano & den Houting, 2022). This movement points to the role of the medical model in autism in stressing the “clinicalness” of symptoms and focusing on impairments, in diminishing the importance of society and professions outside of the clinical sphere, such as in education or the workplace, from acting responsibly and engaging with autism (Doyle, 2020; Hamilton & Petty, 2023; Scott et al., 2019).

The International Classification of Functioning, Disability and Health (ICF) by the WHO and the ICF Children and Youth version (ICF-CY) (WHO, 2001, 2007) could play a key role in bridging the divide between traditional and novel ways of conceptualizing autism. This could result in improving understanding, support, and social awareness of autism, underpinning a shift to more holistic and contemporary perspectives, both pre- and post-diagnosis (Bölte et al., 2021). Employing a biopsychosocial model and comprising codes relevant to both children and adults, the ICF-CY operationalizes an individual’s functioning through 1685 hierarchical codes, classified according to body structures (k = 329) and functions (k = 531) (a person’s somatic and psychological prerequisites and potential), activities (what a person is doing or not doing in their life) and participation (k = 552) (social and societal involvement), and environmental factors (k = 273) (the micro, meso, and macro context). Importantly, the ICF emphasizes that “functioning” should not be confused with impairment. Rather the ICF positions functioning as an etiologically neutral construct, incorporating an individual’s strengths and challenges, as emerging from the interplay between factors including environmental barriers and facilitators. Assessment of functioning practices underpinned by the ICF are motivated by a desire to ascertain support needs, improve participation outcomes, and prevent social exclusion and poor mental health. Conceptualizing functioning in this way delivers important opportunities for individualizing funding, novel approaches to research utilizing enriched client registers, and enhanced data harmonization (Black et al., 2024; Bölte, 2023; Leonardi et al., 2022). The ICF is designed to be accessible to a broad range of professions and applicable across settings, being compatible with paradigms seeking to dimensionalize rather than categorize human behavior (e.g. Research Domain Criteria and transdiagnostics; Fletcher-Watson, 2022; Insel et al., 2010) and salutogenic approaches (e.g. positive psychology and neuro-affirmation; Bertilsdotter Rosqvist et al., 2023; Seligman & Csikszentmihalyi, 2000). Ethical principles are fundamental in operationalizing the ICF framework, and drive value-based assessment practices, including offering opportunities for personal choice, respecting personal values and autonomy, welcoming their participation, and learning from lived experience (Bickenbach, 2012; WHO, 2001). The ICF framework has international credibility, being authorized by the WHO and is endorsed by all its 191 member states representing high-, middle-, and low-income countries, and is also recommended by many national authorities (Bölte, 2023; Leonardi et al., 2022).

Criticisms of the ICF have included that it is too comprehensive and has limited alignment with current diagnostic practices. However, the comprehensiveness of the ICF allows the tailoring of assessments to individual profiles, with most assessments drawing from a significantly smaller subset of ICF codes. In addressing this criticism, the WHO and the ICF Research Branch defined a standardized rigorous procedure for generating ICF Core Sets, that is selections, or shortlists of ICF codes most relevant to specific conditions (Selb et al., 2015). While developing Core Sets may initially appear to contradict the philosophy of the ICF which includes diagnostic neutrality, their intention is to enhance the clinical utility of the ICF and adoption by professionals less familiar with the framework. Importantly, employing the Core Sets in assessing an individual’s functioning does not negate the opportunity to include additional codes as needed. According to the ICF Core Set Branch, developing a Core Set should follow a three-phased, multi-method, and multi-perspective scientific process comprising four preparatory studies: an empirical multi-center study, a systematic literature review, a qualitative study, and an expert survey (Selb et al., 2015). Subsequently, an international consensus conference is held where the collective evidence from the preparatory studies is condensed to form the Core Sets. While the ICF includes codes on up to four levels of increasing functioning detail, Core Sets are usually restricted to the second level of ICF code detail. Once developed, these initial Core Sets require validation in practice. Core Sets have currently been generated for about 30 diagnoses, of which 12 are published in peer-reviewed journals (Tofani et al., 2023), among them attention deficit hyperactivity disorder (ADHD; Bölte et al., 2018), depression (Cieza, Chatterji, et al., 2004), bipolar disorder (Vieta et al., 2007), sleep disorders (Stucki et al., 2008), and cerebral palsy (Schiariti et al., 2015).

In 2016, the initial Core Sets for autism based on ICF-CY codes were adopted and published 3 years later (Bölte et al., 2019) based on the preparatory research and expert consensus outlined above (de Schipper et al., 2015, 2016; Mahdi, Albertowski et al., 2018; Mahdi, Viljoen et al., 2018). A comprehensive and brief Core Set was generated in line with the recommended standardized procedure for Core Sets and previous ICF Core Set developments. A comprehensive Core Set should contain as few ICF codes as possible but as many as necessary to describe the functioning of persons with a certain condition across the entire lifespan, independent of sex or other possible moderators or mediators. A common brief Core Set is a selection of ICF codes from the comprehensive ICF Core Set containing the most typical and significant codes. For autism, the development of three age-appropriate brief Core Sets was also deemed meaningful for research and practice: a preschool set (ages 0–5 years), a school-age set (ages 6–16 years), and an older adolescent and adult set (ages ⩾17 years). The process generated 111 ICF codes in the comprehensive Core Set for autism: 1 body structure, 20 body functions, 59 activities/participation, and 31 environmental factors, 6.5% of the totality of ICF codes and with more than half of the codes reflecting participation and environmental factors (Bölte et al., 2019). The common brief Core Set comprises 60 codes, and the brief age-appropriate Core Sets include 73 codes in the preschool version, 81 in the school-age version, and 79 in the older adolescent and adult version. The age-appropriate brief ICF Core Sets for autism are of intermediate lengths, between the common brief and comprehensive sets.

According to the rigorous protocol for ICF Core Set development, the final step of construction is validation and implementation of the initial Core Sets (Cieza, Ewert, et al., 2004; Grill & Stucki, 2011), a continuous process that is likely to come with revisions, based on empirical studies; changes of diagnostic concepts and zeitgeist; prolonged clinical experience in working with the Core Sets; proof of feasibility in practice; and stakeholder involvement, acceptability, and feedback. The validation and implementation phase is less standardized and has taken different forms and addressed multiple purposes in previous Core Set developments (Kus et al., 2011; Mueller et al., 2010; Sabariego et al., 2013). This article presents the first revision of the initial ICF Core Sets for autism, derived from validation and implementation research and activities, and stakeholder feedback between 2019 and 2023.

## Methods

Ethical approval was sought from the Swedish Ethical Review Authority and the University Sheffield’s Ethics Committee for those parts of the study that required approval. There was community involvement regarding the study design and decision-making within the piloting work of the ICF platform in the United Kingdom outlined below (see also Crowson et al., 2023). In addition, both spontaneous stakeholder feedback directed to the researchers and feedback explicitly collected in the pilot studies (see below) were recorded and were instrumental in generating candidate ICF codes for the ICF Core Sets for autism revision.

### Delphi-like technique

Information from different sources was integrated within a Delphi-like process to form the first revision of the initial ICF Core Sets. The Delphi method is a structured and iterative communication technique relying on the opinions and feedback of a group of experts (Dalkey & Helmer, 1963), employed in different areas of research, services, and guideline development, including recent work in autism (Frazão et al., 2022; Kerns et al., 2023; O’Hagan et al., 2023). The approach is flexible, and no universal guidelines exist, although common components are the identification of a research question, the definition of criteria for the selection of experts, a data collection phase with rounds of opinions, and final decision-making. This study’s prerequisites did not match the format for a typical Delphi process. For example, there was an a priori-defined objective as part of an ongoing ICF Core Set development, a well-defined task to complete using evidence from a multitude of sources, and a limited number of qualified experts available to be included in the process. Therefore, several elements of a typical Delphi technique were either foregone or modified to best serve the study purpose. Thus, our method can be best described as Delphi-like using *bricolage* (Pratt et al., 2022), meaning combining different, primarily qualitative, analytic processes for the purpose of tailoring a method to suit the requirements of the circumscribed research project. The latter aligns with recommendations for Delphi techniques, mainly ensuring that selected procedures align with study objectives, as long as they are plausible (Keeney et al., 2006).

The expert group was purposefully composed of 13 members (all authors; 7 females, 6 males) representing five high-income countries (Sweden, Germany, United Kingdom, Australia, Austria) from two WHO regions (European and Western Pacific), and four professions (psychologist, physician, occupational therapist, linguist) who contributed data, experience, and perspectives in different parts of the study. In addition to the core expert group, additional practitioners contributed to discussions and gave feedback, particularly in Sweden. The inclusion of experts in the core group was based on their combined autism and ICF expertise; their knowledge of the autism ICF Core Sets; involvement in previous or ongoing autism studies applying the autism ICF Core Sets; or their experience of ICF implementation, ICF policymaking, or knowledge of or engagement in the international use of the ICF. The group’s professional experience varied between 3 and 27 years, their experience of autism research or practice between 3 and 27 years, and their experience of the ICF between 1 and 15 years. Thus, the expert group was characterized by a balance of experienced and less experienced members in relevant study domains and of common size for Delphi methods (Hussler et al., 2011; Trevelyan & Robinson, 2015). Group members were not anonymous, but neither they nor their roles were necessarily known to the other group members.

There were no specified numbers or standardized rounds across the experts. Still, there was an open, iterative process over the 4 years facilitated by the first author (S.B.) where subgroups of the expert panel participated and contributed. There were no attrition or non-response issues, although the members’ involvement in the process varied within and between members. The Delphi-like process was guided and driven by collective evidence from international empirical validation/linking studies of the autism ICF Core Sets, stakeholder feedback, and qualitative data from the development and pilot studies of the autism ICF Core Sets platform. The final decisions about adding or removing ICF codes from the initial autism Core Sets were made by consensus of five experts (S.B., E.Z., E.W., L.A., J.H.) who had been extensively involved in the revision process over a longer period. These experts also investigated if adding new codes was formally possible, that is, whether content was ICF codable, if new evidence could be subsumed under existing codes, and the possible effects of removing existing codes. They also examined the candidate ICF codes in relation to the results from the previous four preparatory studies (de Schipper et al., 2015, 2016; Mahdi, Albertowski et al., 2018; Mahdi, Viljoen et al., 2018).

### International research on the ICF autism Core Sets

We examined existing research exploring the validity of the autism ICF Core Sets to identify potential indications that codes needed to be added or removed in revised versions of the initial Core Sets to better capture the functioning of autistic individuals. Included research was published between 2019 and 2023 and examined the validity of the initial Core Sets in various settings (clinic, education, employment, community, cross-cultural) and in relation to various situations or actions (assessment, support, Covid-19) by ICF “linking.” Linking is a technique that allows the calculation of the percentage of study contents covered by the Core Sets codes, providing an indication of the Core Sets’ ability to capture factors pertinent to functioning in autism across settings and situations. Linking is a standard procedure in ICF research, Core Set development, and validation, where findings from empirical studies are coded to the ICF classification system according to standardized rules (Cieza et al., 2005, 2019). We identified and analyzed the results of nine studies: five qualitative (Black et al., 2019; Fridell et al., 2022; Lundin et al., 2021; Thompson et al., 2021; Viljoen et al., 2019) and four reviews (D’Arcy et al., 2023; Hayden-Evans et al., 2022; Leifler et al., 2021; Scott et al., 2019). Studies were analyzed by focusing on areas of functioning and ICF codes reported in the research as not being covered by the initial ICF autism Core Sets to provide indications of areas where the initial Core Sets may not be adequately capturing functioning. The overarching generic significance of these functioning areas and codes for autism was then discussed within the Delphi-like technique.

### Stakeholder feedback

Autistic individuals and their relatives were involved in the preparatory studies and processes underlying the initial versions of the autism ICF Core Sets (Bölte et al., 2019). In addition, they were also included in most of the subsequent validation studies listed above. However, within this research, stakeholders had limited opportunity to directly comment or decide on the final selection of codes in the initial ICF autism Core Sets, as the consensus conference resulting in the initial Core Sets included exclusively (non-autistic) clinicians and researchers. Since the publication of the initial Core Sets, members of the expert group, especially the lead author, have received comprehensive feedback from autistic researchers, autistic self-advocates and activists, interest organizations, and other stakeholders regarding the ICF Core Sets. These comments were recorded, and their appropriateness and significance for the composition of the ICF autism Core Sets were analyzed and discussed in the expert group. Stakeholder feedback reached the authors through social media comments (Twitter, Facebook), face-to-face discussions at scientific meetings and community events (especially after presentations of the Core Sets), via e-mail, and during scheduled meetings with stakeholders.

### Development and pilot studies of the autism ICF Core Sets platform

Since the publication of the initial autism ICF Core Sets, ICF codes included in the initial sets have been operationalized and implemented on a cloud-based Internet platform (https://icfcoresets.se/) to enhance and simplify the application of the ICF in autism. The Swedish, English, and German versions of the platform are currently standardized and validated using the revised sets presented here. Between 2021 and 2023, the platform comprising the initial Core Sets was piloted in Sweden and the United Kingdom using qualitative designs (focus groups/individual interviews) to explore user-friendliness, the appropriateness of the operationalization of ICF codes to items, and the clarity of item wording. A total of 33 individuals diagnosed with autism or ADHD (20 United Kingdom, 13 Sweden), 10 relatives of autistic people or those with ADHD (Sweden), and 29 professionals (16 United Kingdom, 13 Sweden) (psychologists, special education teachers) were included. Moreover, in Sweden, quantitative data were collected to preliminarily validate the Core Sets in a case–control design with 43 autistic children, adolescents, and adults and 248 general population participants. Results from these pilot studies are reported elsewhere in detail (Alehagen et al., 2024). Information of relevance for the revisions of the initial autism ICF Core Sets was extracted from these pilot studies and considered by the expert group.

In addition, the process of operationalizing ICF codes to platform items (questions for self- and informant report) and translating the initial Core Sets from Swedish to English and German resulted in multiple sustained and in-depth discussions about the appropriateness of existing codes. Discussions addressed relevant functioning areas not covered by the original autism Core Sets and particularly the fit of specific ICF codes in the brief age-appropriate Core Sets. The conclusions from these discussions were also integrated into the Delphi-like process.

## Results

### Evidence from validation/linking research on initial autism Core Sets

Table 1 provides an overview of the evidence for potential revisions to the ICF autism Core Sets regarding novel or removed codes generated by examining studies that validated the ICF autism Core Sets. Two studies were conducted in the employment context. The qualitative study by Black et al. (2019) interviewed 68 autistic adults, employers, caregivers, researchers, and professionals across the United States, Sweden, and Australia on success factors for employment in autism. Linking the interview content to the ICF revealed an overall high (89%) content coverage by the initial Core Sets. Still, it also yielded that the initial sets did not cover seven body functions, one activity and participation, and three environmental factors. In their systematic review, Scott et al. (2019) linked 36 articles on employment interventions to the ICF. The initial Core Sets covered all functioning areas mentioned in more than 30% of the evaluated studies. Three codes, two from the activities and participation domain, and one environmental factor, mentioned in more than 10%, but less than 30% of the linked articles, were not covered by the initial sets and were considered as candidates during revision of the Core Sets. Leifler et al. (2021) and Thompson et al. (2021) applied the Core Sets in education contexts. Leifler et al.’s (2021) scoping review synthesized 14 studies on the physical, pedagogical, and social learning environment in autism, concluding that the ICF Core Sets for autism largely covered all relevant learning environment areas. Only the outdoor environment was not sufficiently represented in the initial sets. An interview study examining the perspectives of 13 parents of autistic university students receiving peer mentoring by Thompson et al. (2021) demonstrated that the ICF Core Sets for autism covered 97% of codes applied to the interview data. The remaining uncovered contents were personal factors (e.g. ethnicity, gender, age, educational level, coping styles), which are not coded in the ICF and are not part of official Core Sets. Fridell et al. (2022) linked the initial ICF Core Sets to data exploring the lived experiences of 38 autistic individuals, caregivers, and representatives of interest organizations regarding a range of Covid-19-related outcomes, such as daily activities and socialization, education, work, and access to support and parental resources. The ICF Core Sets for autism covered 84% of all linked codes, with 6 codes from the environmental factors domain not covered. Lundin et al. (2021) studied sex/gender differences in autism in relation to functioning across cultures using qualitative expert survey data from 99 professionals representing 31 countries. The Core Sets covered 97% of the content. Only the environmental factor “strangers,” indicating females being at higher risk for victimization in contact with unknown people, was not covered.

**Table 1.** Overview of ICF candidate codes from each source of evidence and decision for first ICF Core Sets for autism revision.

ICF candidate codes	Validation/linking studies	Stakeholder feedback	Development, pilot studies ICF platform	*Decision by Delphi-like technique*
s110 Brain structure			X	C−
b117 Intellectual functions			X	05−
b163 Basic cognitive functions	X			No
b164 Higher level cognitive functions			X	05−
b172 Calculation functions	X			No
b180 Experience of self- and time functions	X			No
b210 Seeing functions	X	X	X	C+, 05+, 616+, ⩾17+
b230 Hearing functions		X	X	C+, 05+, 616+, ⩾17+
b235 Vestibular functions	X			No
b250 Taste function		X	X	C+, 05+, 616+, ⩾17+
b255 Smell functions		X	X	C+, 05+, 616+, ⩾17+
b265 Touch functions			X	05+, 616+, ⩾17+
b270 Sensory functions related to temperature and other stimuli		X		C+, 616+, ⩾17+
b280 Sensation of pain	X	X	X	C+, 05+, 616+, ⩾17+
b298 Other sensory functions			X	C+, 05+, 616+, ⩾17+
d110 Watching			X	616−
d130 Copying			X	⩾17+
d161 Direct attention			X	⩾17+
d166 Reading			X	616+
d170 Writing			X	C−
d220 Undertaking multiple tasks			X	05−
d349 Communication-producing, other specified and unspecified	X			No
d350 Conversation			X	05+
d360 Using communication devices			X	05−, 616+
d440 Fine hand use	X		X	C+, 05+
d475 Driving			X	616+, ⩾17+
d510 Washing oneself			X	05+, ⩾17+
d520 Caring for body parts			X	616+, ⩾17+
d530 Toileting			X	⩾17+
d540 Dressing			X	05+, ⩾17+
d550 Eating			X	616+
d571 Looking after one’s safety			X	05−
d620 Acquisition of goods/services			X	⩾17+
d630 Preparing meals			X	⩾17+
d650 Caring for household objects			X	⩾17+
d660 Assisting others			X	616+, ⩾17+
d730 Relating with strangers			X	⩾17+
d740 Formal relationships			X	616+
d760 Family relationships			X	05−
d770 Intimate relationships			X	⩾17+
d815 Preschool education			X	05+
d830 Higher education			X	⩾17+
d840 Apprenticeship (work preparation)	X			No
d850 Remunerative employment			X	⩾17+
d930 Religion and spirituality			X	C+, 616+, ⩾17+
e130 Products of technology education			X	05−
e135 Products and technology for employment	X			No
e240 Light		X	X	616+, ⩾17+
e250 Sound		X	X	⩾17+
e260 Air quality	X			No
e298 Natural environment and human-made changes to environment, other specified	X			No
e320 Friends			X	05+
e345 Strangers	X			No
e350 Domestic animals	X	X	X	C+, 616+, ⩾17+
e398 Support and relationships, other specified	X			No
e420 Individual attitudes of friends			X	05+, 616+, ⩾17+
e425 Individual attitudes of acquaintances, peers, colleagues, neighbors, and community members	X		X	C+, 05+, 616+, ⩾17+
e440 Individual attitudes of personal care providers and personal assistants		X	X	C+, 05+, 616+, ⩾17+
e455 Individual attitudes other professionals		X	X	05+, ⩾17+
e545 Civil protection services, systems, and policies	X			No
e555 Associations and organizational services, systems, and policies	X			No
e560 Media services, systems, policies			X	616+
e565 Economic services, systems, and policies	X			No
e590 Labor employment services, systems, policies			X	05−, 616−

C+ = added to comprehensive Core Set; C− = removed from comprehensive Core Set; 05+ = added to age-appropriate 0–5 years Core Set; 05− = removed from age-appropriate 0–5 years Core Set; 616+ = added to age-appropriate 6–16 years Core Set; 616− = removed from age-appropriate 6–16 years Core Set; 17+ = added to age-appropriate ⩾17 years Core Set; ⩾17− = removed from age-appropriate ⩾17 years Core Set; No = no change introduced to any Core Set; ICF: International Classification of Functioning, Disability and Health.

Three of the examined linking studies did not provide evidence for potential revisions, mainly owing to study design, but underpinned the generic validity of the initial sets. Viljoen et al. (2019) compared parent/caregiver perceptions of environmental factors in South Africa and Sweden using the initial ICF autism Core Sets. The authors focused on the frequency and content of codes, predicting that environmental factors would most frequently be reported as barriers to functioning in South Africa. However, only three categories differed qualitatively between South Africa and Sweden, all covered by the initial sets, indicating no need for revisions. The reviews by D’Arcy et al. (2023) and Hayden-Evans et al. (2022) examined how well scales, such as the Vineland Adaptive Behavior Scales-3 or the Adaptive Behavior Assessment System-3, commonly used in autism in different age groups, cover the autism ICF Core Set codes. Due to the design of the study, using the Core Sets as the gold standard for content validation, it generated no evidence for revisions of the autism ICF Core Sets but concluded that commonly used scales largely lack environmental factors of functioning covered in the ICF Core Sets, and instead rely heavily on body functions and activities only.

### Stakeholder feedback

Input from stakeholders came from Sweden, Germany, France, Norway, Denmark, Austria, the United Kingdom, the Netherlands, the United States, and Australia. A total of 39 comments and queries were collected via personal correspondence, tweets, and e-mails. They came predominantly from autistic adults who were researchers, self-advocates, or activists. The feedback concerned both indications of some missing codes for body functions and environment in the initial comprehensive Core Set and a multitude of body functions, activities and participation, and environment codes existing in the initial comprehensive Core Set but missing in the age-appropriate Core Sets. Regarding missing codes in the initial comprehensive Core Set, feedback was quite consistent regarding areas of basic sensory functions (seeing, hearing, taste, smell, pain), religion/spirituality, attitudes from peers, colleagues, neighbors, and personal caregivers, and the role of domestic animals (dogs, cats). See Table 1 for a summary of ICF code candidates for the Core Sets revisions derived from stakeholder feedback.

### Development and pilot studies of the ICF Core Sets platform

See Table 1 for a summary of candidate codes identified for potential addition or removal from the initial ICF autism Core Sets resulting from the development and pilot of the ICF autism Core Sets platform. Operationalization of the initial comprehensive and age-appropriate Core Sets into self- and informant-rating items resulted in many discussions among the involved researchers and clinicians. The most appropriate wording to reflect the intended function in a broadly comprehendible way, the necessary number of items, the logical order of presentation of items, and the best translation of items from Swedish to English and German was debated. Several of these discussions during the development process were of relevance for the revision of the initial Core Sets. First, there was consensus that the body structure code “structure of the brain” should be omitted for several reasons. Especially as it is broad and difficult to meaningfully operationalize, potentially stig-matizing, and likely to not contribute added value to overall functional assessment or support planning. Also, “writing” was suggested to be removed, as it did not emerge as a sufficiently significant code for any of the age-appropriate brief Core Sets. Second, researchers and clinicians agreed that the comprehensive Core Sets are most likely to be applied in research and practice that examines functioning over the lifetime rather than current behavior. However, examining current behavior when applying ICF is rather the rule than the exception. For this reason, the significance of the age-appropriate Core Sets being specific to age and development should be stressed and their contents elaborated. In particular, several activities and participation, and environment items included in the initial age-appropriate Core Sets were impossible or of limited value to collect within the specific age groups. For example, the initial brief set for autism for children aged 0–5 years (Bölte et al., 2019) included items such as household, economy, school, work, and intimate relationships, while the ⩾17 years initial Core Set included items such as play. Therefore, there was agreement (a) that some additional codes needed to be removed to depict age-specific functional repertoires, but also (b) that many codes from the comprehensive Core Sets needed to be added to the age-appropriate Core Sets to enable stand-alone use from the comprehensive set. Suggestions were foremost to add several activities and participation and environment codes to all three age-appropriate Core Sets and add body functions codes to the 6–16 and ⩾17 years Core Sets. In addition, removing some codes from age-appropriate Core Sets, particularly the 0–5 years version, was recommended.

The pilot studies of the ICF Core Sets platform described elsewhere in detail (Alehagen et al., 2024; Crowson et al., 2023) corroborated parts of the evidence from the platform development phase and the feedback from stakeholders described above. Even though participants welcomed the assessment and found it feasible, caregivers completing the operationalized Core Sets comm-ented on missing and superfluous areas of functioning when filling out age-appropriate Core Sets. Particularly, these participants reacted to items in the 0–5 years brief version and recommended removing them. Further, both autistic individuals and relatives commented on missing functioning areas, for example, touch functions, sensory functions related to temperature and other, driving, self-care, housekeeping, light and sound conditions, and attitudes in the social environment.

### Added and removed ICF codes after the Delphi-like procedure

Table 1 shows which ICF codes were finally removed or added from the comprehensive and age-appropriate ICF Core Sets for autism based on the Delphi-like method. Twelve candidate codes were rejected and did not lead to any changes. A total of 12 ICF codes were added to the comprehensive: six body functions (b210, b230, b250, b255, b280, and b298), three activity and participation codes (d440, d815, and d930), and three environmental factors (e350, e425, and e440). One body structure code (s110) and one activities and participation code (d170) were removed. Seventeen codes were added to the 0–5 years Core Sets: seven body functions (b210, b230, b250, b255, b265, b280, and b298), five activities and participation (d350, d440, d510, d540, and d815), and five environmental factors (e320, e420, e425, e440, and e455); eight codes were removed: two body functions (b117 and b164), four activities and participation (d220, d360, d571 and d760), and two environmental factors (e130 and e590). To the 6–16 years Core Set, 22 codes were added: eight body functions (b210, b230, b250, b255, b265, b270, b280, and b298), eight activities and participation (d166, d360, d475, d520, d550, d660, d740, and d930), and six environmental factors (e240, e350, e420, e425, e440, and e560). One activity and participation code (d110) and one environmental factor (e590) were removed. We added 31 codes to the ⩾17 years Core Set: 8 body functions (b210, b230, b250, b255, b265, b270, b280, and b298), 16 activities and participation (d130, d161, d475, d510, d520, d530, d540, d620, d630, d650, d660, d730, d770, d830, d850, and d930), and 7 environmental factors (e240, e250, e350, e420, e425, e440, e455). The common brief ICF Core Sets for autism were not revised.

### The revised ICF Core Sets for autism

The updated revised comprehensive ICF Core Sets for autism, and the three corresponding age-appropriate Core Sets (0–5, 6–16, and ⩾17 years) are shown in Appendix 1–4. The revised comprehensive Core Set now contains 121 ICF codes (7.2% of all ICF-CY codes), 26 body functions, 61 activities and participation, and 34 environmental factors. The revised 0–5 years age-appropriate Core Set contains 82 ICF codes: 24 body functions, 30 activities and participations, and 28 environmental factors. The revised 6–16 years Core Set now includes 101 ICF codes: 26 body functions, 43 activities and participation, and 32 environmental factors. Finally, the older adolescents and adults Core Sets (⩾17 years) comprise, after revision, 110 ICF codes: 26 from body functions, 50 from activities and participation, and 34 from environmental factors.

## Discussion

We present here the first revision of the initial ICF Core Sets for autism that were adopted in 2016 and published in 2019 (Bölte et al., 2019). Since publication of these initial Core Sets for autism, there has been significant development in the field of autism research and a shift in thinking among the autistic community, making a revision of these initial Core Sets advisable and timely. In revising the initial Core Sets, we sought to exploit and integrate relevant information collected over half a decade from various sources, including ICF Core Set for autism validation and linking studies, stakeholder feedback, experiences and data from the operationalization and implementation of the Core Sets on an online assessment platform, and piloting of the platform. A Delphi-like process was used to guide the revision. The comprehensive Core Set and the three age-appropriate Core Sets for autism were revised and enlarged, with only a few codes removed.

The linking/validation studies of the comprehensive Core Set and pilot studies of the operationalized Core Sets using the online assessment tool generally indicated good to excellent coverage of functioning in autism for different settings and life domains and satisfaction with the assessment by various informants and user groups. Still, autistic individuals strongly desired the inclusion of additional areas of functioning. These primarily concerned various domains of sensory functioning which their relatives and professionals also endorsed. We added ICF body function codes for seeing, hearing, taste, smell, and pain sensation to the comprehensive Core Set, a total of six new ICF second-level codes, to address this feedback. Although these additions mean extending the sets, we believe they are meaningful for a variety of reasons. First, these changes were supported not only by the collected new evidence presented here but also by most of the preparatory studies of the initial ICF Core Sets (de Schipper et al., 2016; Mahdi, Albertowski et al., 2018; Mahdi, Viljoen et al., 2018), although not included following the consensus conference, which comprised only researchers and health care professionals (Bölte et al., 2019). Second, and importantly, while co-creation with autistic people is advocated for in autism research (Fletcher-Watson et al., 2019; Rodríguez Mega, 2023), the development of the initial ICF Core Sets did not sufficiently involve the voices and primary perspectives of autistic people, which the expert group of this study deemed a priority to consider in this revision. Third, robust evidence from different study designs indicates that hypersensitivity and hyposensitivity are integral to autism phenotypes (Neufeld et al., 2021; C. E. Robertson & Baron-Cohen, 2017; A. E. Robertson & Simmons, 2015). Thus, it might be surprising that more ICF codes representing sensory processing were not included in the initial versions. The former decision to limit the number of ICF codes reflecting sensory issues was made because it is still unclear whether sensory processing alterations experienced by autistic people are indeed “sensory” in nature, that is, representing altered physiological sensation in peripheral receptors and primary sensory cortical areas only, or rather “perception,” referring to how the incoming primary sensory information is organized, and consciously experienced in the brain (Coren, 2003; C. E. Robertson & Baron-Cohen, 2017). Moreover, it is debated how environments might affect sensation and perception (Hadad & Yashar, 2022). Perception and related psychological constructs (e.g. thought, memory and attention functions, learning) were already largely covered in the initial comprehensive Core Set, as were environmental factors like sound and smell. Still, after stakeholder feedback and empirical studies presented here, it appears clear that scientific ambiguity should not stand in the way of maximizing the practical use of the ICF, which conceptionally is non-etiological and meant to prioritize target population utility. The Delphi-like process deemed that including more sensory function ICF codes is crucial for the revised comprehensive Core Sets for autism to (a) generate high identification with the ICF code selection, and (b) offer broad options to evaluate experiences in the sensory/perception field of autism that facilitate or hinder functioning and are therefore important for support and participation planning. In addition to body function codes for sensory processing, the ICF activities and participation codes for fine hand use and religion/spirituality were included based on indications from validation studies, experiences from the ICF Core Set platform, and all four previous preparatory studies (de Schipper et al., 2016; Mahdi, Albertowski et al., 2018; Mahdi, Viljoen et al., 2018). Including fine hand use is also supported by autism research particularly indicating alterations in picking up, manipulating, and releasing objects in young autistic children (Sacrey et al., 2014). Generally, motor challenges and developmental coordination disorder are quite prevalent in autism (Licari et al., 2020; Miller et al., 2024). Spirituality and religion have been reported by some autistic people to be of importance for affirmation and acceptance of their difficulties (Liu et al., 2014).

Two new ICF codes were included in the comprehensive Core Sets that reflect attitudes toward autism or autistic people expressed by acquaintances, peers, colleagues, neighbors, personal care providers, or assistants. Even these had been previously indicated by the preparatory studies of the initial Core Sets (de Schipper et al., 2016; Mahdi, Albertowski et al., 2018; Mahdi, Viljoen et al., 2018), and were now endorsed again by experiences from the development and pilot studies of the ICF platform, on one hand, and validation/linking studies of the initial Core Sets or stakeholder feedback, on the other hand. These additions to the comprehensive Core Set expand on the significance of the social facilitators and barriers in the environment for functioning in autism. They are both in line with calls from the neurodiversity paradigm, stressing the significance of social factors for disability in autism (den Houting, 2019) and the increasing body of research highlighting the important role that the broader community has to play in supporting the functional outcomes of autistic individuals (Dickter et al., 2020; Kuzminski et al., 2019; White et al., 2019).

Finally, the code domestic animals is a newly included code to the comprehensive ICF autism Core Set. It had not been indicated previously in any of the preparatory studies that led to the initial Core Sets but was consistently indicated in the recent Core Set validation/linking studies, the development and piloting of the ICF Core Sets platform, and by stakeholders. Assistive, therapeutic, and companion animals have been shown in several studies to contribute to enhanced participation and well-being in autism and are likely to play a vital role in the functioning of some autistic individuals (Atherton et al., 2023; Carlisle et al., 2020; Germone et al., 2019).

In addition to enlarging the comprehensive ICF Core Set for autism, a multitude of items from the initial and the revised comprehensive Core Sets were added to the age-appropriate brief Core Sets. The most significant reasons for these modifications were: (a) to transfer new ICF codes from the revised comprehensive Core Sets to the relevant age-appropriate brief sets, and (b) to make the age-appropriate brief sets more useful in practice to be used stand-alone and independently from the comprehensive Core Sets without important functional information loss. Concerning the latter, our Delphi-like process indicated that future use of age-appropriate ICF Core Sets is the most likely scenario in research and practice. This is first because most researchers and practitioners predominantly work with circumscribed age groups (infants/young children; children/adolescents; adults) which aligns with the age-appropriate ICF autism Core Sets, and, second, because a blanket approach to administering the comprehensive Core Set frequently leads to superfluous ICF codes (e.g. adult relevant codes in infants) that might cause confusion, irritation, or be unnecessarily time-consuming. In addition, the common brief ICF autism Core Set might lack much functional content experienced as essential by stakeholders. Therefore, we did not update the common brief ICF autism Core Set, owing to our decision to focus on the age-appropriate brief Core Sets, and do not recommend their use until further notice. Another consequence of prioritizing the age-appropriate Core Sets is that the revised Comprehensive Core Set for autism only includes ICF codes that appear in any of the age-appropriate sets, which is different from the initial comprehensive Core Set which also had unique additional codes.

However, limitations to generating age-appropriate selections of ICF codes were also encountered when revising the age-appropriate autism Core Sets. Particularly, age-appropriate selections are difficult for developmentally intense age groups (e.g. infancy/early childhood) and for age groups that include environment-related transitions (e.g. education to work–life). For example, several ICF codes included in the 0–5 years brief autism Core Set, while important in assessing functioning in children aged 4 or 5 years, may not apply (yet) to younger ages, such as verbal communication or basic self-care functions. We also needed to include ICF codes in the ⩾17 years Core Sets, which are likely not to apply to all below age 20 years, especially in high-income countries, such as those related to employment or independent living. Indeed, though the initial Core Set development involved multiple countries across WHO regions, this revision included almost exclusively data from high-income countries. Thus, in keeping with ICF Core Set development methodology, validations of the revised Core Sets should be conducted at a global level to ensure that revisions are culturally sensitive and are reflective of the experiences of autistic individuals globally.

We removed two ICF codes from the initial comprehensive Core Set, namely “writing” and “brain structure.” Writing was removed as it did not emerge as a sufficiently significant code for any of the age-appropriate brief Core Sets in the piloting, and therefore did not qualify any longer for the Comprehensive Set, according to our decision that the Comprehensive Set should not comprise codes that are not part of any of the age-appropriate Core Set. Brain structure, the only body structure in the initial Core Set, was deemed expendable because it could be potentially stigmatizing and not useful for functioning assessment and support planning in autism when using the ICF. While autism is a neurodevelopmental condition, which was the motivation to include the brain structure code in the initial Core Sets version, there is no single structural brain alteration or other neurological marker sensitive or specific in autism, indicative of any specific support or sensitive to intervention (Frye et al., 2019; Parellada et al., 2023). Some ICF codes being part of the previous age-appropriate Core Sets were removed, as they were not deemed to be sufficiently informative or specific to the given age group or sufficiently covered by other codes (e.g. labor/employment in 0–5 and 6–16 years). There were several instances where we refrained from including some additional codes in the Core Set revisions, despite evidence emerging within our study. Some were not autism specific but were instead pandemic or crisis specific such as “civil protection services.” Others were covered by an overlapping ICF code, although from another domain, such as “strangers” as an environmental factor (behaviors of strangers) not being included in addition to “relating with strangers” as an activity.

This is one of the first ever conducted ICF Core Set revisions, and while there is a standard protocol for the initial development of ICF Core Sets (Selb et al., 2015), no gold standard exists for revisions. We collected different forms of evidence on the validity and feasibility of the initial ICF autism Core Sets (Bölte et al., 2019) and sorted and condensed them in a Delphi-like process. This process was a compromise to make most sense of the multi-source aggregated data and evidence in the eyes of an active group of international ICF in autism researchers and clinicians, of whom still rather few exist. There are also other methodological limitations, such as most of the evidence being used originating from high-income countries and no autistic researchers being part of the expert panel. Moreover, our collection of stakeholder feedback was largely an informal process. While it conferred several advantages, such as a lower threshold for diverse spontaneous contributions, it also required own initiative. The latter was perhaps limiting autism community stakeholder feedback, as those who did not reach out to the authors may have had different concerns or valuable contributions, which were missed.

These aspects need to be kept in mind and improved when designing the methods for future revisions of the ICF autism Core Sets. Despite these shortcomings, we believe the revisions are based on sufficiently consistent and solid evidence and decision-making, are widely comprehensible, and improve the initial version’s validity and acceptability, adding value to ICF Core Sets use in research and practice.

## Appendix

**Appendix 1.** Second-level ICF codes included in the comprehensive ICF Core Set for autism.

b114 Orientation functions	d310 Communicating with—receiving—spoken messages
b117 Intellectual functions	d315 Communicating with—receiving—nonverbal messages
b122 Global psychosocial functions	d330 Speaking
b125 Dispositions and intra-personal functions	d331 Pre-talking
b126 Temperament and personality functions	d335 Producing nonverbal messages
b130 Energy and drive functions	d350 Conversation
b134 Sleep functions	d360 Using communication devices and techniques
b140 Attention functions	d440 Fine hand use
b144 Memory functions	d470 Using transportation
b147 Psychomotor functions	d475 Driving
b152 Emotional functions	d510 Washing oneself
b156 Perceptual functions	d520 Caring for body parts
b160 Thought functions	d530 Toileting
b164 Higher level cognitive functions	d540 Dressing
b167 Mental functions of language	d550 Eating
b210 Seeing functions	d570 Looking after one’s health
b230 Hearing functions	d571 Looking after one’s safety
b250 Taste function	d620 Acquisition of goods and services
b255 Smell functions	d630 Preparing meals
b265 Touch functions	d640 Doing housework
b270 Sensory functions related to temperature and other stimuli	d650 Caring for household objects
b280 Sensation of pain	d660 Assisting others
b298 Other sensory functions	d710 Basic interpersonal interactions
b330 Fluency and rhythm of speech functions	d720 Complex interpersonal interactions
b760 Control of voluntary movement functions	d730 Relating with strangers
b765 Involuntary movement functions	d740 Formal relationships
d110 Watching	d750 Informal social relationships
d115 Listening	d760 Family relationships
d130 Copying	d770 Intimate relationships
d132 Acquiring information	d815 Preschool education
d137 Acquiring concepts	d820 School education
d140 Learning to read	d825 Vocational training
d145 Learning to write	d830 Higher education
d155 Acquiring skills	d845 Acquiring, keeping, and terminating a job
d160 Focusing attention	d850 Remunerative employment
d161 Directing attention	d860 Basic economic transactions
d163 Thinking	d870 Economic self-sufficiency
d166 Reading	d880 Engagement in play
d175 Solving problems	d910 Community life
d177 Making decisions	d920 Recreation and leisure
d210 Undertaking a single task	d930 Religion and spirituality
d220 Undertaking multiple tasks	d940 Human rights
d230 Carrying out daily routine	e110 Products or substances for personal consumption
d240 Handling stress and other psychological demands	e115 Products and technology for personal use in daily living
d250 Managing one’s own behavior	
e125 Products and technology for communications	e425 Individual attitudes of acquaintances, peers, colleagues, neighbors, and community members
e130 Products and technology for education	e430 Individual attitudes of people in positions of authority
e240 Light	e440 Individual attitudes of personal care providers and personal assistants
e250 Sound	e450 Individual attitudes of health professionals
e310 Immediate family	e455 Individual attitudes of other professionals
e315 Extended family	e460 Societal attitudes
e320 Friends	e465 Social norms, practices, and ideologies
e325 Acquaintances, peers, colleagues, neighbors, and community members	e525 Housing services, systems, and policies
e330 People in positions of authority	e535 Communication services, systems, and policies
e340 Personal care providers and personal assistants	e550 Legal services, systems, and policies
e350 Domestic animals	e560 Media services, systems, and policies
e355 Health professionals	e570 Social security services, systems, and policies
e360 Other professionals	e575 General social support services, systems, and policies
e410 Individual attitudes of immediate family members	e580 Health services, systems, and policies
e415 Individual attitudes of extended family members	e585 Education and training services, systems, and policies
e420 Individual attitudes of friends	e590 Labor and employment services, systems, and policies

ICF: International Classification of Functioning, Disability and Health.

**Appendix 2.** The second-level ICF codes included in the age-appropriate ICF Core Set for autism 0–5 years.

b114 Orientation functions	d350 Conversation
b122 Global psychosocial functions	d440 Fine hand use
b125 Dispositions and intra-personal functions	d510 Washing oneself
b126 Temperament and personality functions	d530 Toileting
b130 Energy and drive functions	d540 Dressing
b134 Sleep functions	d550 Eating
b140 Attention functions	d570 Looking after one’s health
b144 Memory functions	d710 Basic interpersonal interactions
b147 Psychomotor functions	d720 Complex interpersonal interactions
b152 Emotional functions	d815 Preschool education
b156 Perceptual functions	d880 Engagement in play
b160 Thought functions	d920 Recreation and leisure
b167 Mental functions of language	e110 Products or substances for personal consumption
b210 Seeing functions	e115 Products and technology for personal use in daily living
b230 Hearing functions	e125 Products and technology for communication
b250 Taste function	e240 Light
b255 Smell functions	e250 Sound
b265 Touch functions	e310 Immediate family
b270 Sensory functions related to temperature and other stimuli	e315 Extended family
b280 Sensation of pain	e320 Friends
b298 Other sensory functions	e325 Acquaintances, peers, colleagues, neighbors, and community members
b330 Fluency and rhythm of speech functions	e330 People in positions of authority
b760 Control of voluntary movement functions	e340 Personal care providers and personal assistants
b765 Involuntary movement functions	e355 Health professionals
d110 Watching	e360 Other professionals
d115 Listening	e410 Individual attitudes of immediate family members
d130 Copying	e415 Individual attitudes of extended family members
d132 Acquiring information	e420 Individual attitudes of friends
d137 Acquiring concepts	e425 Individual attitudes of acquaintances, peers, colleagues, neighbors, and community members
d155 Acquiring skills	e430 Individual attitudes of people in positions of authority
d160 Focusing attention	e440 Individual attitudes of personal care providers and personal assistants
d161 Directing attention	e450 Individual attitudes of health professionals
d210 Undertaking a single task	e455 Individual attitudes of other professionals
d230 Carrying out daily routine	e460 Societal attitudes
d240 Handling stress and other psychological demands	e465 Social norms, practices, and ideologies
d250 Managing one’s own behavior	e550 Legal services, systems, and policies
d310 Communicating with—receiving—spoken messages	e570 Social security services, systems, and policies
d315 Communicating with—receiving—nonverbal messages	e575 General social support services, systems, and policies
d330 Speaking	e580 Health services, systems, and policies
d331 Pre-talking	e585 Education and training services, systems, and policies
d335 Producing nonverbal messages	

ICF: International Classification of Functioning, Disability and Health.

**Appendix 3.** The second-level ICF codes included in the age-appropriate ICF Core Set for autism 6–16 years.

b114 Orientation functions	d510 Washing oneself
b117 Intellectual functions	d520 Caring for body parts
b122 Global psychosocial functions	d530 Toileting
b125 Dispositions and intra-personal functions	d540 Dressing
b126 Temperament and personality functions	d550 Eating
b130 Energy and drive functions	d570 Looking after one’s health
b134 Sleep functions	d571 Looking after one’s safety
b140 Attention functions	d660 Assisting others
b144 Memory functions	d710 Basic interpersonal interactions
b147 Psychomotor functions	d720 Complex interpersonal interactions
b152 Emotional functions	d730 Relating with strangers
b156 Perceptual functions	d740 Formal relationships
b160 Thought functions	d750 Informal social relationships
b164 Higher level cognitive functions	d760 Family relationships
b167 Mental functions of language	d820 School education
b210 Seeing functions	d880 Engagement in play
b230 Hearing functions	d920 Recreation and leisure
b250 Taste function	d930 Religion and spirituality
b255 Smell functions	e110 Products or substances for personal consumption
b265 Touch functions	e115 Products and technology for personal use in daily living
b270 Sensory functions related to temperature and other stimuli	e125 Products and technology for communication
b280 Sensation of pain	e130 Products and technology for education
b298 Other sensory functions	e240 Light
b330 Fluency and rhythm of speech functions	e250 Sound
b760 Control of voluntary movement functions	e310 Immediate family
b765 Involuntary movement functions	e315 Extended family
d115 Listening	e320 Friends
d130 Copying	e325 Acquaintances, peers, colleagues, neighbors, and community members
d132 Acquiring information	e330 People in positions of authority
d137 Acquiring concepts	e340 Personal care providers and personal assistants
d140 Learning to read	e350 Domestic animals
d145 Learning to write	e355 Health professionals
d155 Acquiring skills	e360 Other professionals
d160 Focusing attention	e410 Individual attitudes of immediate family members
d161 Directing attention	e415 Individual attitudes of extended family members
d163 Thinking	e420 Individual attitudes of friends
d166 Reading	e425 Individual attitudes of acquaintances, peers, colleagues, neighbors, and community members
d175 Solving problems	e430 Individual attitudes of people in positions of authority
d177 Making decisions	e440 Individual attitudes of personal care providers and personal assistants
d210 Undertaking a single task	e450 Individual attitudes of health professionals
d220 Undertaking multiple tasks	e455 Individual attitudes of other professionals
d230 Carrying out daily routine	e460 Societal attitudes
d240 Handling stress and other psychological demands	e465 Social norms, practices, and ideologies
d250 Managing one’s own behavior	e535 Communication services, systems, and policies
d310 Communicating with—receiving—spoken messages	e550 Legal services, systems, and policies
d315 Communicating with—receiving—nonverbal messages	e560 Media services, systems, and policies
d330 Speaking	e570 Social security services, systems, and policies
d350 Conversation	e575 General social support services, systems, and policies
d360 Using communication devices and techniques	e580 Health services, systems, and policies
d470 Using transportation	e585 Education and training services, systems, and policies
d475 Driving	

ICF: International Classification of Functioning, Disability and Health.

**Appendix 4.** The second-level ICF codes included in the age-appropriate ICF Core Set for autism ⩾17 years.

b114 Orientation functions	d650 Caring for household objects
b117 Intellectual functions	d660 Assisting others
b122 Global psychosocial functions	d710 Basic interpersonal interactions
b125 Dispositions and intra-personal functions	d720 Complex interpersonal interactions
b126 Temperament and personality functions	d730 Relating with strangers
b130 Energy and drive functions	d740 Formal relationships
b134 Sleep functions	d750 Informal social relationships
b140 Attention functions	d760 Family relationships
b144 Memory functions	d770 Intimate relationships
b147 Psychomotor functions	d820 School education
b152 Emotional functions	d825 Vocational training
b156 Perceptual functions	d830 Higher education
b160 Thought functions	d845 Acquiring, keeping, and terminating a job
b164 Higher level cognitive functions	d850 Remunerative employment
b167 Mental functions of language	d860 Basic economic transactions
b210 Seeing functions	d870 Economic self-sufficiency
b230 Hearing functions	d880 Engagement in play
b250 Taste function	d910 Community life
b255 Smell functions	d920 Recreation and leisure
b265 Touch functions	d930 Religion and spirituality
b270 Sensory functions related to temperature and other stimuli	d940 Human rights
b280 Sensation of pain	e110 Products or substances for personal consumption
b298 Other sensory functions	e115 Products and technology for personal use in daily living
b330 Fluency and rhythm of speech functions	e125 Products and technology for communication
b760 Control of voluntary movement functions	e130 Products and technology for education
b765 Involuntary movement functions	e240 Light
d130 Copying	e250 Sound
d132 Acquiring information	e310 Immediate family
d155 Acquiring skills	e315 Extended family
d160 Focusing attention	e320 Friends
d161 Directing attention	e325 Acquaintances, peers, colleagues, neighbors, and community members
d166 Reading	e330 People in positions of authority
d175 Solving problems	e340 Personal care providers and personal assistants
d177 Making decisions	e350 Domestic animals
d210 Undertaking a single task	e355 Health professionals
d220 Undertaking multiple tasks	e360 Other professionals
d230 Carrying out daily routine	e410 Individual attitudes of immediate family members
d240 Handling stress and other psychological demands	e415 Individual attitudes of extended family members
d250 Managing one’s own behavior	e420 Individual attitudes of friends
d310 Communicating with—receiving—spoken messages	e425 Individual attitudes of acquaintances, peers, colleagues, neighbors, and community members
d315 Communicating with—receiving—nonverbal messages	e430 Individual attitudes of people in positions of authority
d330 Speaking	e440 Individual attitudes of personal care providers and personal assistants
d350 Conversation	e450 Individual attitudes of health professionals
d360 Using communication devices and techniques	e455 Individual attitudes of other professionals
d470 Using transportation	e460 Societal attitudes
d475 Driving	e465 Social norms, practices, and ideologies
d510 Washing oneself	e525 Housing services, systems, and policies
d520 Caring for body parts	e535 Communication services, systems, and policies
d530 Toileting	e550 Legal services, systems, and policies
d540 Dressing	e560 Media services, systems, and policies
d570 Looking after one’s health	e570 Social security services, systems, and policies
d571 Looking after one’s safety	e575 General social support services, systems, and policies
d620 Acquisition of goods and services	e580 Health services, systems, and policies
d630 Preparing meals	e585 Education and training services, systems, and policies
d640 Doing housework	e590 Labor and employment services, systems, and policies

ICF: International Classification of Functioning, Disability and Health.
